# A New, Safe, and Effective Technique for Percutaneous Insertion of a Peritoneal Dialysis Catheter

**DOI:** 10.3390/jcm13092618

**Published:** 2024-04-29

**Authors:** Andrzej Jaroszyński, Jarosław Miszczuk, Marcin Jadach, Stanisław Głuszek, Wojciech Dąbrowski

**Affiliations:** 1 Collegium Medicum, Jan Kochanowski University of Kielce, 25-369 Kielce, Poland; j.miszczuk@op.pl; 2 Department of Nephrology, Specjalistyczny Szpital Powiatowy w Stalowej Woli, 37-450 Stalowa Wola, Poland; marcinjadach83@gmail.com; 3 Department of General, Oncological and Endocrinological Surgery, Collegium Medicum, Jan Kochanowski University of Kielce, 25-369 Kielce, Poland; sgluszek@wp.pl; 4 Department of Anaesthesiology and Intensive Therapy, Medical University of Lublin, 20-059 Lublin, Poland; w.dabrowski5@yahoo.com

**Keywords:** Veress needle, intravascular catheter, percutaneous catheter placement, peritoneal dialysis, peritoneal catheter, outcomes

## Abstract

A properly functioning peritoneal catheter is an essential element of effective peritoneal dialysis (PD). Currently, there are three techniques available for PD catheter placement, which include open surgery, laparoscopic surgery, and percutaneous catheter placement (PCP). Currently, no particular catheter placement approach has been proven with certainty to provide superior outcomes. We present a new modified PCP method with the use of the Veress needle covered with an intravascular catheter (IC) and preliminary clinical results of PD catheter placements with this new technique. The endpoints used in the study were 1-year technical survival of the catheter, and the incidence of early (1 month) mechanical as well as infection complications. The catheter was implanted in 24 patients. The catheter survival rate was 100%; however, in two cases, the catheters were removed due to complications not associated with PD treatment. No early mechanical complications such as bleeding, hematoma, perforations, internal organ damage, exit site leaks, or hernia in the place of insertion were observed. Similarly, no early infectious complications were observed. During the 1-year follow-up, no catheter migration occurred. Our results showed that the new PCP technique is a safe and easy procedure that minimizes the occurrence of both mechanical and infectious complications and ensures good catheter survival.

## 1. Introduction

Peritoneal dialysis (PD) is a renal replacement therapy with some potential additional benefits over hemodialysis, including better preservation of residual renal function, better hemodynamic stability, and improved quality of life. It requires minimal infrastructure yet provides similar survival rates compared to hemodialysis and is cost-effective. In addition, PD is the preferred method for patients awaiting kidney transplantation [1,2,3,4,5,6,7]. 

Proper catheter placement is a key element for successful peritoneal dialysis (PD). At present, there are three techniques available for PD catheter placement, which include open surgery, laparoscopic surgery, and percutaneous catheter placement (PCP) [1,2,3,4,5]. Currently, there is no consensus on the preferred catheter placement method. Each technique has its own advantages, disadvantages, and complications. The most commonly used methods are surgery techniques. However, both surgery methods should be performed by experienced surgeons and require general anesthesia. The availability of a surgical team may limit the number of procedures performed. Additionally, surgical methods require general anesthesia, which may be associated with potential complications, especially in end-stage renal disease patients co-morbid with advanced chronic heart failure [1,2,3,4]. On the other hand, surgical techniques have an advantage due to the possibility of performing partial omentectomy, omentopexy, or adhesiolysis during the catheter implantation [1,2,3,4,5].

The PCP technique is minimally invasive, and is accessible to nephrologists, which may increase the availability of the procedure. In most cases, PCP can be performed under local anesthesia, saving both cost and time. When fluoroscopic guidance is used, an additional benefit of both minimal invasiveness and avoidance of general anesthesia is the accurate, real-time imaging confirmation of catheter positioning throughout the procedure. The main disadvantage is that PCP cannot be recommended or recommended with great caution to at-risk patients, such as patients with previous abdominal surgery for whom surgery techniques are preferred. Currently, less than 5% of PD catheters are placed by nephrologists [1,6,7]. Although there are some modifications to PCP, commercially available kits are the most commonly used. Commercially available catheter implantation kits contain a needle used to puncture the abdominal cavity and insert a guidewire into the peritoneum. The procedure is performed under ultrasound control. After the insertion of the guidewire, a dilator and the peel-away sheath are advanced over the guidewire into the abdominal cavity, allowing implantation of the catheter [1,2,3,4,5,6,7]. However, the procedure performed in this way may carry a risk of mechanical complications, especially when performed without fluoroscopic control.

We present a new modified PCP method with the use of the Veress needle covered with an intravascular catheter (IC) and preliminary clinical results of PD catheter placements with this new technique.

## 2. Materials and Methods

### 2.1. Patients

Included in the study were all adult patients who qualified for PD treatment at a single center in Stalowa Wola (Poland) from December 2022 to February 2023. All patients gave written consent, and the study was approved by members of the local ethics committees. Exclusion criteria were as follows: uncorrectable bleeding diathesis, abdominal interventions highly increasing the risk of massive adhesions, and other abdominal pathologies precluding proper PD treatment, as recommended by KDIGO [1].

The endpoints used in the study were as follows: 1-year technical survival, and the incidence of early (1 month) mechanical as well as infection complications. Catheter survival rate was defined as a catheter removal within a year, associated exclusively with PD complications.

### 2.2. Procedure

**Figure 1:**
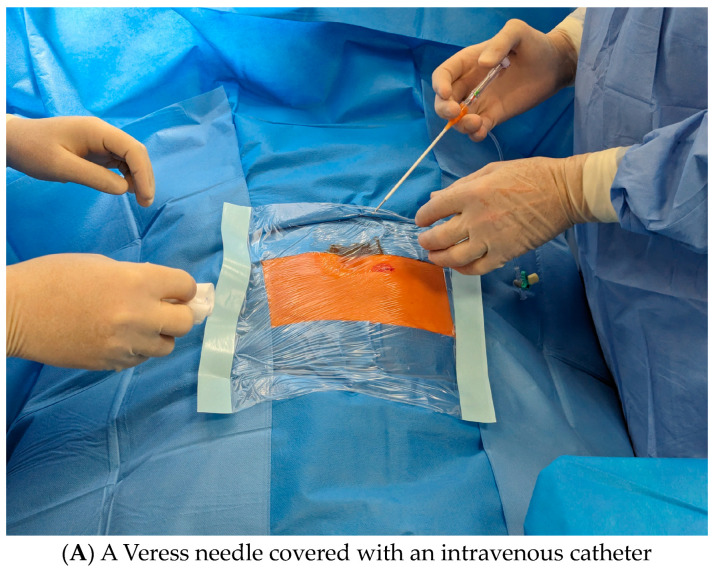

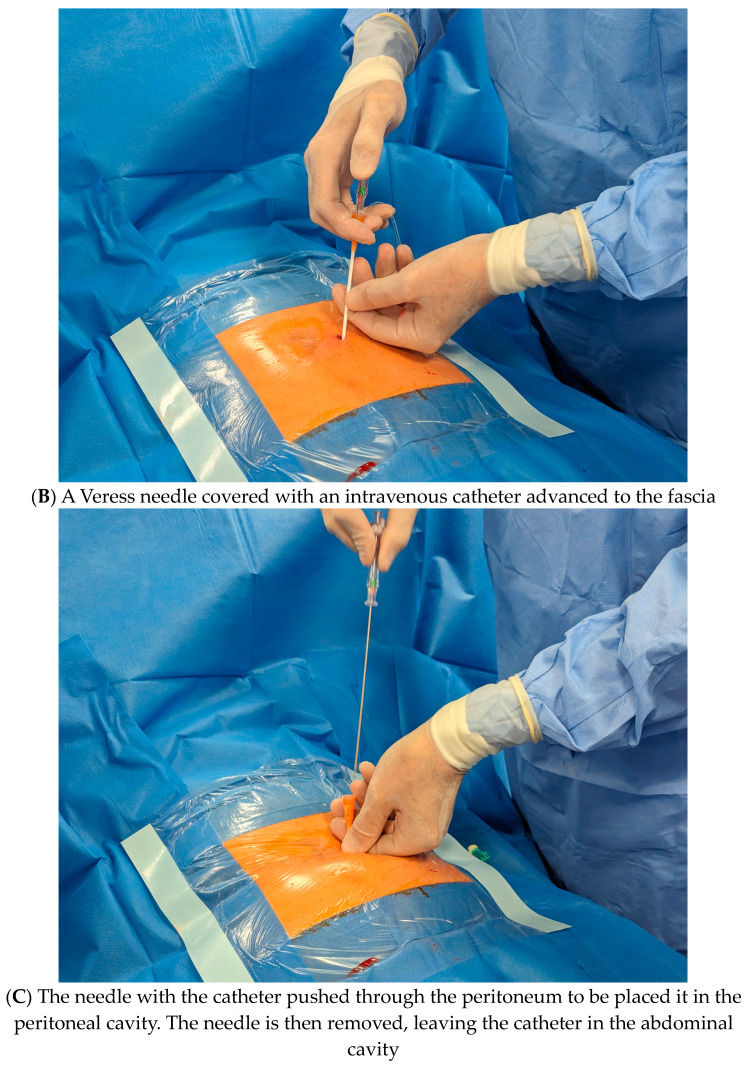

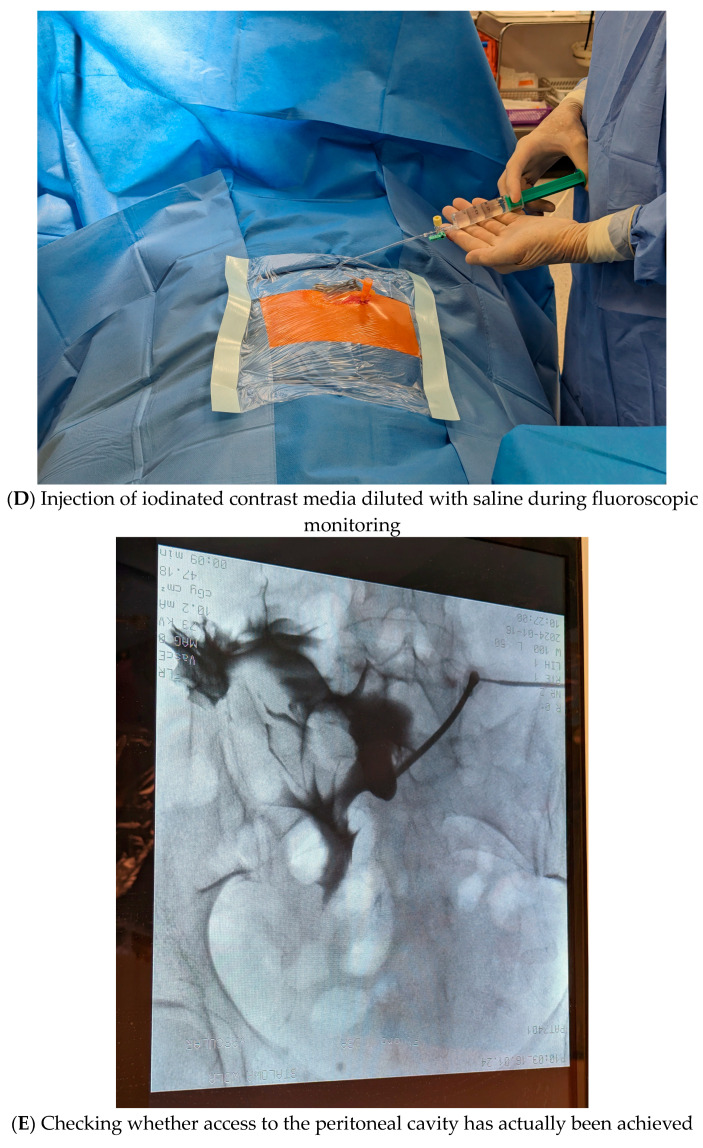

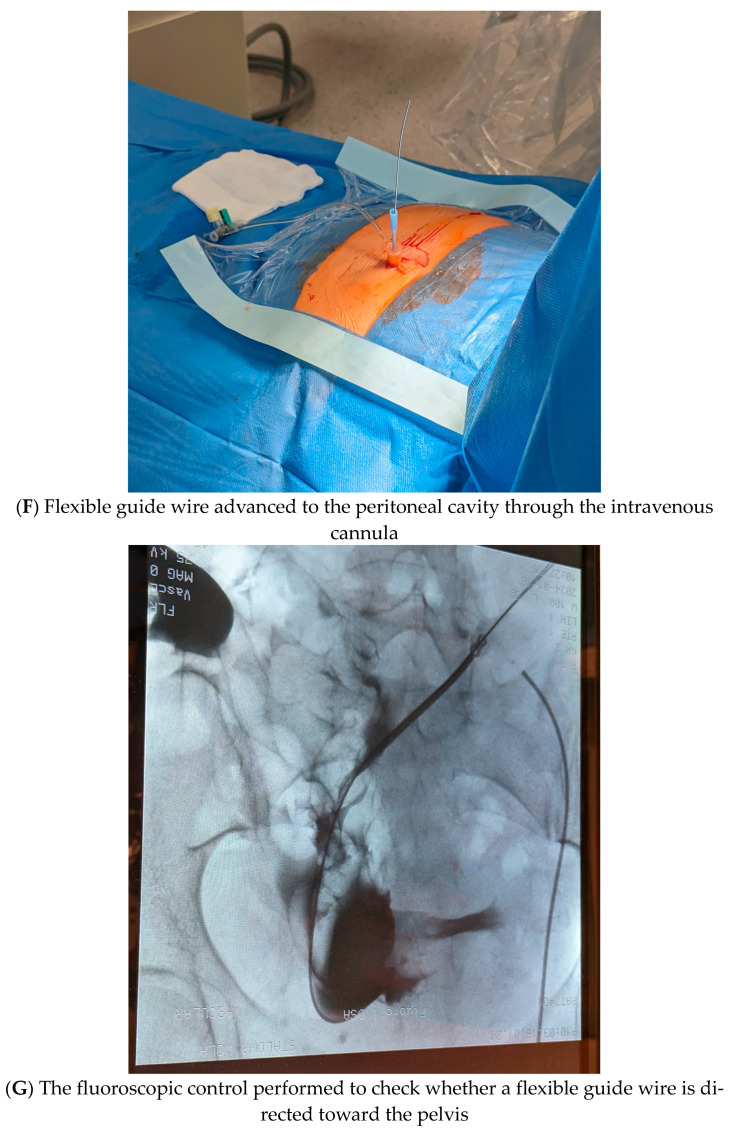

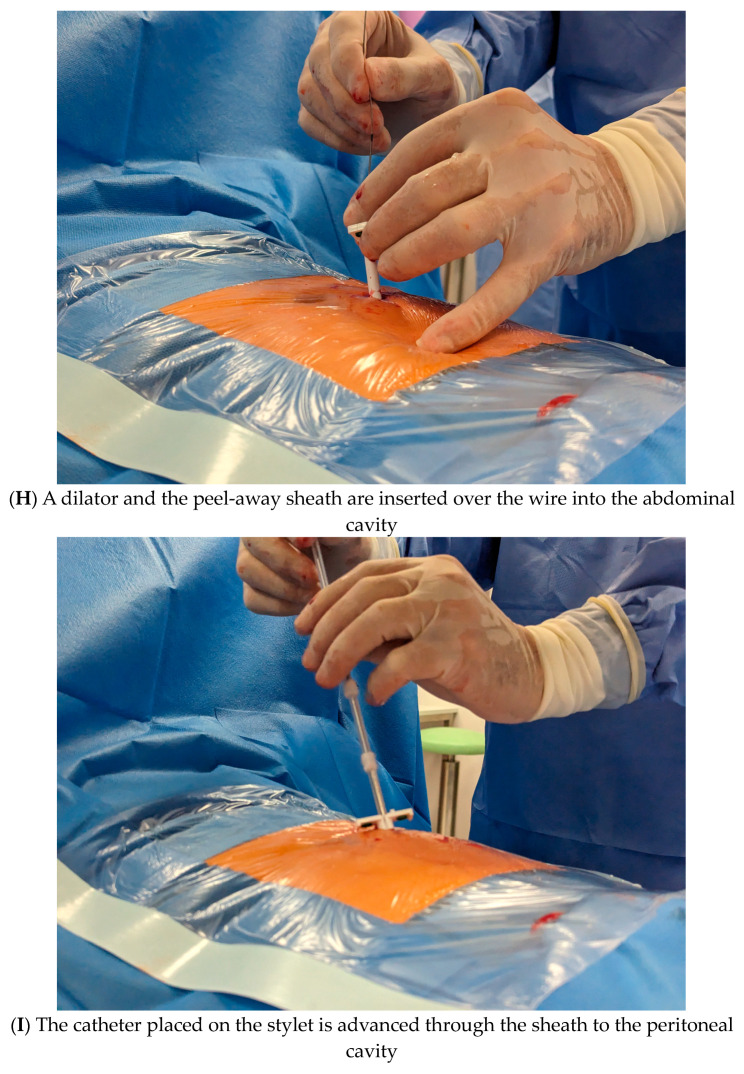

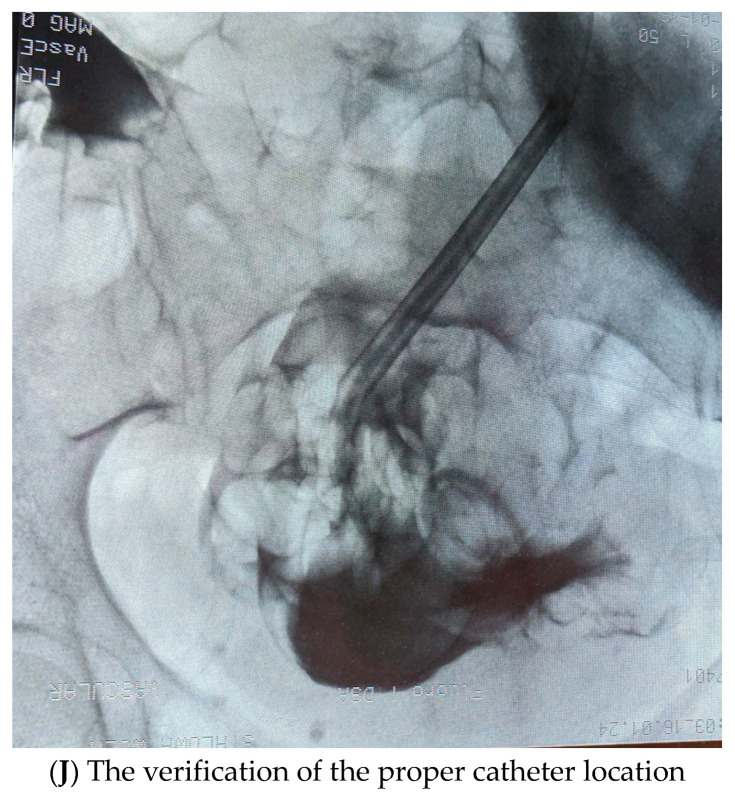
Subsequent stages of PCP with a new method using the Veress needle covered with an intravascular catheter—detailed description in the text.

(1) The pre-operative patient preparation for the PCP was performed according to the KDIGO recommendations [1]. In the pre-operative planning stage, patients were examined while in both supine and sitting positions for visualization of an appropriate exit site, taking into account abdominal folds and the height of the trouser/skirt waistband. In the case of right-handed patients, the exit side is preferred on the left side, while in left-handed patients, it is preferred on the right side.
(2) The evening before the procedure, the patient had an enema, and prophylactic antibiotic therapy consisting of cephazolin (1.0 g intravenous-Biotaksym, Polpharma S.A., Starogard Gdański, Poland) was administered 60 min before the procedure. The bladder was emptied just before the procedure.
(3) After preparing the surgical field and the patient’s skin was anesthetized intradermally with 2% lidocaine (Lignocainum Hydrochloricum, Polfa Warszawa, Warszawa, Poland), a small incision (less than 1 cm but enough to introduce catheter internal Dacron cuff) using triangular scalpel blade 11 was created below and laterally and below to the umbilicus (lateral insertion).
(4) The tissues were then bluntly dilated using a pair of paean forceps so as to reach fascia. A Veress needle (Veress Needle, Grena Ltd., Brentford Middlesex, UK) covered with an intravenous catheter (Radiofocus Introducer, Terumo Corp., Lenven, Belgium) (Figure 1A) was advanced to the fascia (Figure 1B). Next, the needle with the catheter was pushed firmly through the peritoneum to place it in the peritoneal cavity. The needle was then removed, leaving the catheter in the abdomen (Figure 1C). To verify if access to the peritoneal cavity was actually achieved, iodinated contrast media diluted with saline in a 1:1 ratio was injected during fluoroscopic monitoring (Figure 1D,E).
(5) Once peritoneal cavity access was achieved, a flexible guide wire was advanced through the intravenous cannula (Figure 1F). Next, the fluoroscopic control was performed to check whether a flexible guide wire was directed toward the pelvis (Figure 1G). If yes, the intravenous catheter was replaced, and a dilator and the peel-away sheath (Argyle–Peritoneal Dialysis Catheter Kit, Covidien, Mansfield, MA, USA) were advanced over the wire into the abdominal cavity (Figure 1H). Next, a guide wire as well as the introducer of the peel-away sheath were removed, leaving the tubing in the abdomen.
(6) The coiled catheter was then placed on the stylet (double-cuff Tenckhoff coiled catheter—Covidien, Mansfield, MA, USA), advanced through the sheath to the peritoneal cavity (Figure 1I). The catheter tip is placed deeply into the recto-vesical space in men and the recto-uterine space in women, which is the most gravity-dependent region where dialysate fluid accumulates, potentially providing the best drainage of dialysis fluid. To verify the proper location, the fluoroscopic location was used (Figure 1J). The internal catheter cuff was placed next to fascia. Next, a catheter flow test was performed to check the catheter function (300–500 mL of peritoneal fluid was administered and drained).
(7) The tunnel between the place of insertion of the catheter into the peritoneal cavity and the exit site was made subcutaneously using metallic tunneling trochar. The course of the tunnel should be directed laterally and downward, and the distal cuff should be subcutaneously 2.5 cm from the exit side. One skin suture was placed where the catheter was inserted into the abdomen. An X-ray image was taken to document the correct positioning of the catheter.

## 3. Results

Out of the 25 patients with ESRD referred to the nephrology unit for peritoneal catheter implantation, 24 qualified for the PCP technique. One patient was excluded due to previous peritonitis. The group included 13 women and 11 men, aged 34 to 87 years (mean, 67.2 ± 10.4 years). The reasons for ESRD were as follows: diabetes (n = 9), glomerulonephritis (n = 8), unknown/unsure (n = 5), interstitial kidney disease (n = 1), and hypertension (n = 1). A total of 7 of the 24 patients had a history of abdominal surgery (3 patients had cesarean section, 2 had an appendectomy, 1 had a cholecystectomy, and 1 had a hysterectomy). A total of 14 patients had diabetes, 12 had heart failure, and 21 had hypertension. Three patients were switched to PD from hemodialysis. For the remaining patients, PD was their first choice. The mean body mass index was 27.9 ± 4.25.

In one case, PCP was unsuccessful, and a surgical implantation was needed. In all cases, lateral insertion of the catheter through the rectus muscle with catheter cuff below rectus muscle was performed. In three cases, the fluoroscopic control revealed that the peritoneal cavity was not obtained and needed a second puncture. In four patients, a guide wire was not directed downward and required repositioning. The initiation of dialysis was delayed for at least 2 weeks following PCP. The catheter survival rate was 100%; however, in two cases, the catheters were removed due to complications not associated with the PD treatment (recovery of renal function and gynecological complications—endometriosis). No early (1 month) mechanical complications, such as bleeding, hematoma, perforations, internal organ damage, exit site leaks, or hernia in the place of insertion, were observed. Similarly, no early infectious complications were observed. During the 1-year follow-up, no catheter migration occurred. In one patient, an event of transient catheter dysfunction occurred due to clogging by fibrin, which required unclogging the catheter.

## 4. Discussion

The use of a Veress needle covered with an IC to create the cerebrospinal fluid peritoneal shunt was described by Lockhart [8]. However, according to our knowledge, this technique has never been used/described for the PCP.

In our study, in one case, PCP was unsuccessful, and a patient needed a surgical implantation. The reason for the failure was incorrect qualification for PCP of the patient after hysterectomy and radiotherapy, which we were unaware about at the moment of qualification. This confirms that patients at a high risk of adhesions should not be qualified for PCP. This is in line with ISPD guidelines and other studies [1,3,5,9]. 

Our study has shown a 1-year catheter survival rate in all patients; however, in two cases, catheters were removed. In both cases, no technical or infection complications were observed, and the reason for the catheter removal was not associated with PD complications. The debate on the advantages of PD catheter implantation methods is still open. No catheter placement approach has been proven with certainty to provide superior outcomes; however, laparoscopy is recommended by KDIGO [1,5,10,11,12]. There are studies showing that laparoscopy may be superior compared to other techniques [1,3,12]. On the other hand, some studies have revealed that PCP is at least non-inferior compared to both surgical methods [6,11,12,13]. The diversity of the results is probably related to the skills and experience of the operator, patient selection factors, and history of abdominal surgery. Considering our results, they should be deemed promising.

Regarding early infectious complications, we did not observe any such complications. The results pertaining to the relation between the method of catheter placement and infection are divergent. Some studies suggest differences in early infectious complications in favor of PCP [11,13,14,15]; however, others have contested this view [16]. The smaller number of early infections may be due to the less invasive nature of PCP, providing a smaller port of entry for microorganisms [13]. It could be only a matter of debate to what extent the small incision, punctual puncture of the abdomen, the initiation of dialysis delayed for at least 2 weeks following PCP, and lack of leakages contributed to the reduced risk of infections. We also administered a single preoperative dose of prophylactic cefazolin to prevent early infection, as recommended by KDIGO [1], while being aware of the increasing antibiotic resistance of many pathogens [17]. Regardless of the reason, our results show that a new PCP is associated with a low risk of early infectious complications.

Similarly, no early mechanical complications were observed. Recently, growing evidence has shown that PCP is probably non-inferior in regard to mechanical complications compared to surgical methods [3,7,9,13,14,15,18]. Evidence exists that the overall rate of intra-abdominal injury while establishing pneumoperitoneum with the use of a Veress needle is about 0.13% [19]. With high probability, these results can be interpolated to the number of mechanical complications during the PCP with our method. We believe that the use of a Veress needle covered with IC is safer than the use of a sharp needle, even under ultrasonography.

Our patients did not experience catheter migration, resulting in malfunction. We did not perform an X-ray to check the correct positioning of the catheter after one year following its insertion, and not every migration causes catheter dysfunction. The incidence of catheter migration ranges from 5 to 35%, with the vast majority (about 85%) occurring within the first two weeks after catheter insertion [2,13,20,21]. The lack of catheter migrations may be due to several reasons: the catheter tip was placed deeply between the rectum and bladder or uterus, and to verify its proper location, the fluoroscopic method was used. 

All PCPs were performed under local anesthesia. Given that most end-stage renal disease (ESRD) patients belong to classes 3–4 of the ASA score [18] and that the postoperative mortality of ESRD patients under general anesthesia is higher than those without ESRD [18], this can be important, especially in patients with comorbidities.

## 5. Limitations

The main limitation of our study is the small number of patients. The results of our PCP method using a Veress needle covered with IC are, however, highly positive. Thus, we decided to describe our new PCP method and publish our study as a preliminary result, realizing that our study needs to be confirmed in further larger studies.

## 6. Conclusions

The PCP method using a Veress needle covered with an IC is a safe and easy procedure that minimizes the occurrence of both mechanical and infectious complications, and ensures good catheter survival. The procedure can be performed by a trained nephrologist, which may make PD more accessible. However, our results need to be confirmed in further larger studies, comparing the new PCP method with other methods, including open surgery, laparoscopic surgery, and other PCP methods.

## Data Availability

The data associated with this study are presented in the article. Detailed descriptions of each percutaneous insertion of the peritoneal dialysis catheter and clinical data of all patients as well as radiological documentation are available from the corresponding author upon reasonable request for researchers who meet the criteria for access to confidential data.
